# The Prediction of Extended Hospital Length of Stay in Patients After Endoscopic Endonasal Transsphenoidal Surgery for the Resection of Non-Functioning Pituitary Adenomas: A Dual-Center Retrospective Analysis

**DOI:** 10.3390/cancers18101582

**Published:** 2026-05-13

**Authors:** Bibo Gao, Junjian Dai, Xiao Yu, Shilong Cao, Congcong Wu, Changsen Zhu, Bingchan Li, Anquan Shang, Ning Wang, Jianguo Meng

**Affiliations:** 1 Department of Neurosurgery, The First Affiliated Hospital of Kunming Medical University, Kunming 650500, China; gaobibo@ydyy.cn (B.G.); 20240807@kmmu.edu.cn (J.D.); 2 Department of Otolaryngology-Head and Neck Surgery, Shanghai Sixth People’s Hospital Affiliated to Shanghai Jiao Tong University School of Medicine, Shanghai 200030, China; lbc1423@sjtu.edu.cn; 3 Department of Neurosurgery, The First People’s Hospital of Yunnan Province, Kunming 650500, China; caoshilong@stu.kust.edu.cn (S.C.); wucongcong@stu.kust.edu.cn (C.W.); 4 Department of Otolaryngology-Head and Neck Surgery, The First People’s Hospital of Yunnan Province, Kunming 650500, China; 20232236205@stu.kust.edu.cn; 5 Department of Laboratory Medicine, Affiliated Lianyungang Clinical College of Nantong University, Lianyungang 222000, China; shanganquan@ntu.edu.cn; 6 Department of Neurosurgery, Zhuji Affiliated Hospital of Wenzhou Medical University, Zhuji 312000, China; wangning198411@163.com; 7 Department of Neurosurgery, Yancheng Tinghu District People’s Hospital, Yancheng 224000, China

**Keywords:** non-functioning pituitary adenomas, endoscopic endonasal transsphenoidal surgery, extended hospital length of stay, nomogram, risk prediction, perioperative management

## Abstract

Background: Prolonged hospitalization after endoscopic endonasal transsphenoidal surgery for non-functioning pituitary adenomas increases costs and complications. Early identification of high-risk patients is crucial for optimizing perioperative management. Methods: In this dual-center retrospective study of 368 patients, a predictive model was developed using a training cohort (*n* = 268). Prolonged length of stay was defined as ≥75th percentile (≥16 days). LASSO regression selected features from clinical, radiological, and perioperative variables. Independent predictors from multivariable logistic regression were dichotomized via ROC analysis and integrated into a nomogram. Model performance was assessed internally and validated externally (*n* = 100). Results: Six independent predictors were identified: age > 50 years, vertical tumor diameter > 17.8 mm, anteroposterior diameter > 20.5 mm, transverse diameter > 17.8 mm, anesthesia duration > 194 min, and systolic blood pressure > 119 mmHg. The nomogram showed moderate but reproducible discrimination (AUC = 0.762 in training; 0.750 in validation). Calibration and decision curve analysis confirmed good fit and clinical utility. Conclusion: We developed and validated a practical nomogram predicting prolonged hospitalization risk using readily available perioperative variables. This tool may assist individualized risk stratification and perioperative planning in comparable clinical settings, with potential implications for patient flow and resource utilization.

## Simple Summary

Endoscopic endonasal transsphenoidal surgery is a standard treatment for non-functioning pituitary adenomas, but the length of postoperative hospitalization can vary considerably among patients. Prolonged hospital stay is clinically important because it may increase healthcare costs, delay recovery, and place additional pressure on medical resources. Early identification of patients at higher risk of extended hospitalization may help improve perioperative management and discharge planning. In this dual-center retrospective study, we developed and externally validated a nomogram to predict the risk of prolonged hospital stay using routinely available clinical and perioperative factors. Older age, larger tumor size, longer anesthesia duration, and higher systolic blood pressure were associated with increased risk. This model may provide a practical tool for individualized risk stratification and perioperative planning in patients undergoing surgery for non-functioning pituitary adenomas.

## 1. Introduction

Endoscopic endonasal transsphenoidal surgery (EETS) is now widely regarded as the standard surgical approach for pituitary adenoma resection, owing to its minimally invasive nature, superior anatomical visualization, and favorable neurological and endocrinological outcomes [1,2,3]. Despite continuous refinements in surgical techniques and perioperative care, postoperative recovery after EETS remains heterogeneous, and a substantial proportion of patients experience an unexpectedly prolonged hospital length of stay (LOS) [4]. Such variability in recovery not only reflects underlying differences in patient and tumor characteristics but also poses significant challenges to perioperative planning and health system efficiency.

Extended hospital length of stay (ELOS) following pituitary surgery has consistently been associated with increased healthcare expenditure, higher rates of hospital-acquired complications, delayed functional recovery, and diminished patient satisfaction [5]. From an institutional perspective, extended hospitalization further constrains bed availability and operating room turnover, particularly in high-volume neurosurgical centers. Consequently, the ability to identify patients at high risk for ELOS at an early stage represents a clinically meaningful target for perioperative optimization, individualized discharge planning, and rational resource allocation [6,7].

A growing body of the literature has investigated determinants of LOS after transsphenoidal surgery, implicating factors such as advanced age, tumor size and invasiveness, surgical complexity, perioperative complications, and postoperative endocrine disturbances [8,9]. However, existing studies are characterized by substantial methodological heterogeneity, frequently limited by single-center designs, small sample sizes, or an emphasis on isolated predictors rather than integrated risk assessment [10]. Moreover, many prior analyses rely on conventional regression approaches without robust feature selection or external validation, which restricts their generalizability and limits translation into routine clinical decision-making [11,12]. Most previous studies have focused on identifying correlates of prolonged LOS rather than developing a disease-specific and externally validated individualized prediction tool for early perioperative risk stratification.

In parallel, predictive modeling strategies incorporating machine learning-based feature selection and intuitive visualization tools have gained increasing traction in neurosurgical research [13,14,15]. These approaches enable the integration of multidimensional clinical, radiological, and perioperative variables to generate individualized risk estimates that are readily interpretable at the bedside. Nevertheless, despite the clinical relevance of LOS as a perioperative outcome, validated prediction models specifically addressing ELOS after EETS for non-functioning pituitary adenomas (NFPAs) remain scarce, and external validation across independent cohorts is uncommon. Accordingly, the present study sought to develop and externally validate a clinically applicable nomogram for predicting prolonged hospital LOS in patients undergoing EETS for NFPA resection. Using a dual-center retrospective cohort, we applied the machine learning for data-driven feature selection, followed by multivariable logistic regression to identify independent predictors of extended hospitalization. Model performance was rigorously evaluated in terms of discrimination, calibration, and clinical utility, with external validation to assess robustness and generalizability.

## 2. Materials and Methods

### 2.1. Study Design and Patient Population

This dual-center retrospective study analyzed clinical data from patients who underwent endoscopic endonasal transsphenoidal surgery (EETS) at the First Affiliated Hospital of Kunming Medical University and the Zhuji Affiliated Hospital of Wenzhou Medical University between July 2023 and September 2025. The study protocols were approved by the institutional ethics review boards of both hospitals (numbers: 2025KM11089 and ZJ202520318) and adhered to the Declaration of Helsinki. Patients were eligible for inclusion if they had pituitary tumors confirmed by magnetic resonance imaging (MRI) and subsequently underwent surgical resection. All included cases were histopathologically confirmed as NFPAs. Furthermore, records were only reviewed for patients who had completed a preoperative visual field assessment using a Humphrey Field Analyzer (Carl Zeiss-Meditec, Dublin, CA, USA). Additionally, tumor diameters in all directions (vertical, anteroposterior, and transverse) were precisely measured via preoperative MRI. Exclusion criteria included functioning pituitary adenomas, recurrent tumors, incomplete clinical records, or perioperative mortality. All included tumors were histopathologically confirmed pituitary adenomas according to the World Health Organization classification [16].

### 2.2. Definition of Postoperative Outcome

The primary outcome was ELOS, defined as the duration from the date of surgery to discharge. ELOS was dichotomized based on the 75th percentile of the length of stay within the training cohort, consistent with established methodologies for analyzing skewed healthcare data. This approach is consistent with previous studies analyzing skewed LOS distributions [17,18,19]. Patients meeting or exceeding this threshold were classified into the ELOS group (coded as 1), while those below the threshold were categorized as the non-ELOS group (coded as 0). Discharge decisions were made by the treating neurosurgical team according to routine institutional practice at each participating center. In general, patients were considered eligible for discharge when they had stable neurological status, no new or progressive visual or neurological deficits requiring inpatient management, stable vital signs, no evidence of active cerebrospinal fluid leakage or intracranial infection, stable fluid-electrolyte and endocrine status, adequate oral intake and mobilization, acceptable postoperative nasal cavity or wound condition, and no need for continued inpatient monitoring or intervention. The ELOS threshold used in this study was applied only for statistical outcome classification and was not used as a clinical discharge criterion.

### 2.3. Candidate Predictors and Data Collection

A comprehensive array of candidate variables was extracted [20], including demographic data such as age, sex, and body mass index (BMI). Tumor-related characteristics derived from MRI included vertical, front-to-back, and left-to-right diameters. Perioperative variables encompassed surgery and anesthesia durations (minutes), while comorbidities included hypertension, diabetes mellitus, heart disease, and stroke. These comorbidities were selected because they were the most consistently and reliably documented baseline systemic diseases across both institutional datasets and could therefore be incorporated in a standardized manner. Other systemic comorbidities, such as chronic pulmonary, hepatic, or thyroid disease, were not uniformly recorded across both centers and therefore were not included in the primary analysis. Vital signs and laboratory parameters consisted of systolic and diastolic blood pressure, fasting glucose, triglycerides, HDL, LDL, albumin, hemoglobin, and platelet counts. Hematological indices included neutrophil, lymphocyte, and monocyte counts. Endocrine profiles covered cortisol and ACTH levels at three time points (08:00, 16:00, 24:00), alongside TSH, T3, T4, FT3, FT4, and growth hormone (GH) levels. Postoperative variables were not incorporated into the primary candidate set because the aim of this study was to develop an early perioperative risk stratification model based on variables available before or during surgery. Although selected postoperative complications were recorded, their event frequency was very low, and other key postoperative variables, including formal invasion grading, extent of resection, and endocrine outcomes, were not systematically available across both centers.

### 2.4. Model Development and Risk Factor Identification

Candidate predictors were first entered into a least absolute shrinkage and selection operator (LASSO) logistic regression model in the training cohort. The optimal penalty parameter was selected by 10-fold cross-validation using the minimum mean cross-validated binomial deviance criterion (lambda.min = 0.0351). Variables with non-zero coefficients at lambda.min were retained as candidate predictors for subsequent multivariable analysis. These retained variables were then entered into a multivariable logistic regression model to identify independent predictors of ELOS, and odds ratios (ORs) with 95% confidence intervals (CIs) were calculated. To improve clinical interpretability and bedside applicability, receiver operating characteristic (ROC) curve analyses were subsequently performed for each independent continuous predictor in the training cohort, and the optimal cutoff value was determined using the maximum Youden index [21]. Continuous predictors were then dichotomized according to these cutoffs and re-entered into a multivariable logistic regression model to construct the primary clinically applicable prediction model. Although dichotomization may lead to information loss and reduced statistical efficiency, the primary purpose of the nomogram was to provide a simple, interpretable, and easily applicable perioperative risk-stratification tool rather than a purely algorithmic prediction model optimized only for maximal numerical discrimination. Threshold-based predictors may facilitate rapid bedside interpretation, risk communication, and individualized discharge planning in routine clinical practice. Therefore, the dichotomized model was retained as the primary clinically oriented model. To evaluate whether model performance was materially affected by dichotomization, a continuous-predictor model using the same variables was additionally constructed as a sensitivity analysis.

### 2.5. Nomogram Construction

A nomogram was constructed on the basis of the final multivariable logistic regression model using the dichotomized independent predictors identified in the training cohort. Each predictor was assigned a point value proportional to its regression coefficient, and the total score was mapped to the predicted probability of ELOS. A supplementary nomogram based on continuous predictors was also generated as part of the sensitivity analysis [22].

### 2.6. Model Performance and Validation

Model performance was evaluated in both the training cohort and the external validation cohort. Discrimination was assessed using the area under the ROC curve (AUC) and the concordance index (C-index). DeLong’s test was used to compare the discriminative performance of the nomogram with that of individual predictors [23]. Calibration was assessed using calibration plots with 1000 bootstrap resamples and the Hosmer-Lemeshow goodness-of-fit test [24]. In addition, calibration was quantified using the calibration intercept and calibration slope, with ideal values of 0 and 1, respectively. In the training cohort, these values reflect apparent calibration, whereas in the validation cohort they provide a quantitative assessment of external calibration. Clinical utility was evaluated by decision curve analysis (DCA), which was used to estimate the net benefit of the nomogram across a range of threshold probabilities in both cohorts.

### 2.7. Statistical Analysis

Statistical analyses were performed using R software (version 4.2.1). Continuous variables were tested for normality using the Shapiro–Wilk test; normally distributed data were expressed as mean ± standard deviation and compared using Student’s *t*-test. Categorical variables were presented as frequencies and percentages and compared using the chi-square test. Multivariable analysis results were reported as odds ratios (ORs) with 95% confidence intervals (CIs). In the training cohort, 68 ELOS events were available for the final model with six predictors, corresponding to an events-per-variable ratio greater than 10, which is generally considered acceptable for logistic regression modeling. All statistical tests were two-sided, and a *p*-value < 0.05 was considered statistically significant.

## 3. Results

### 3.1. Population Baseline Characteristics

A total of 368 patients with histopathologically confirmed NFPAs were included. Among them, 268 patients from the First Affiliated Hospital of Kunming Medical University comprised the training cohort, while 100 patients from the Zhuji Affiliated Hospital of Wenzhou Medical University constituted the external validation cohort (Table 1). The mean length of stay (LOS) was 12.50 ± 4.24 days in the training cohort and 12.22 ± 4.16 days in the validation cohort, with no significant difference observed between cohorts (t = −0.59, *p* = 0.556). Baseline demographic characteristics, tumor dimensions, perioperative variables, comorbidities, and laboratory parameters are summarized in Table 1. No significant differences were identified between the two cohorts across key baseline variables (all *p* > 0.05), confirming good comparability between datasets. Within the training cohort, the 75th percentile of LOS was 16 days. Based on this threshold, 68 patients (25.4%) were classified into the ELOS group, whereas 200 patients (74.6%) were assigned to the non-ELOS group. With respect to postoperative events, no cerebrospinal fluid leakage and diabetes insipidus occurred in either cohort, and intracranial infection was observed only rarely (3 cases in the training cohort and 1 case in the validation cohort). Because of the very low event frequency, these complications were not suitable for meaningful statistical modeling. In addition, tumor invasion grading, extent of resection, and postoperative endocrine outcomes were not systematically available across both centers.

### 3.2. Identification of Independent Risk Factors for Elos

**Figure 1:**
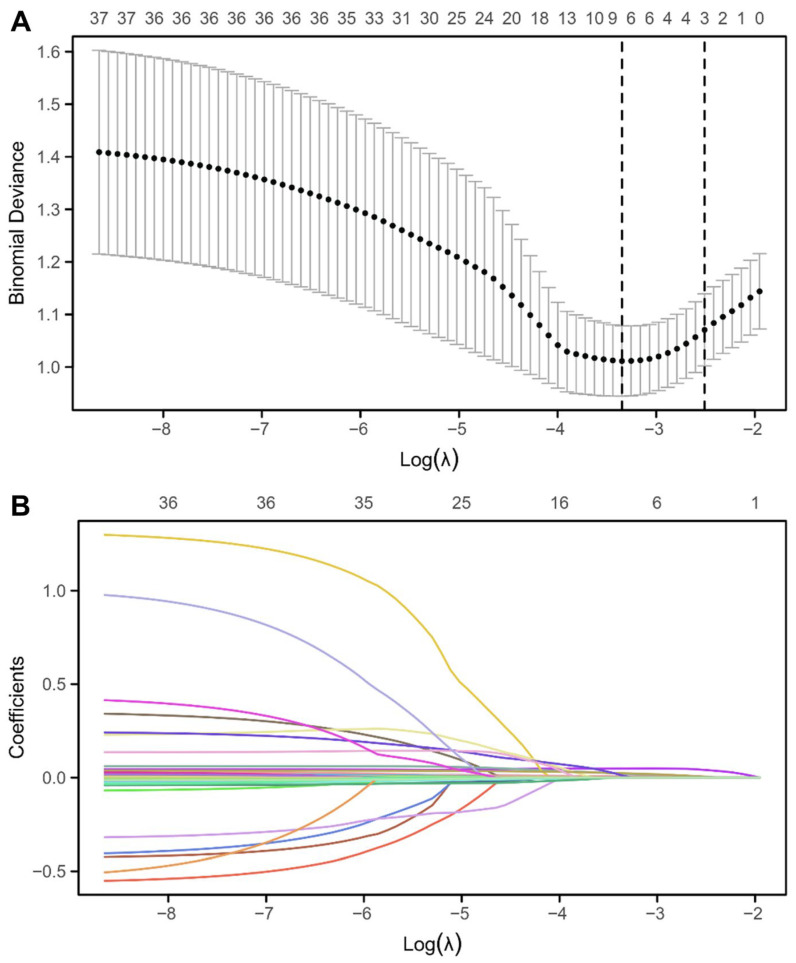
LASSO regression coefficient profiles and cross-validation curve for variable selection in predicting extended length of stay. (**A**) Cross-validation curve showing mean binomial deviance (with error bars representing standard error) versus log(λ). The optimal λ (vertical dashed line) corresponds to the minimum cross-validated error, resulting in eight non-zero coefficient variables. (**B**) Coefficient profiles of candidate variables as a function of the LASSO regularization parameter (λ), illustrating the shrinkage of coefficients toward zero with increasing λ (colorful lines). Vertical dashed line indicates the optimal λ selected by 10-fold cross-validation. Abbreviation: LASSO, least absolute shrinkage and selection operator.

LASSO regression identified candidate predictors associated with ELOS in the training cohort. Coefficient shrinkage plots demonstrated progressive penalization of variables, while 10-fold cross-validation identified the optimal λ corresponding to the minimum prediction error (Figure 1). Eight predictors with non-zero coefficients were retained, including age, tumor dimensions (vertical, anteroposterior, and transverse diameters), anesthesia duration, systolic blood pressure, blood glucose, and ACTH8. All candidate predictors identified by LASSO regression were subsequently entered into a multivariable logistic regression model. Six variables were independently associated with extended length of stay (ELOS), including age, vertical tumor diameter, anteroposterior tumor diameter, transverse tumor diameter, anesthesia duration, and systolic blood pressure (Table 2). Increasing age was associated with a higher risk of ELOS (odds ratio [OR], 1.03 per year; 95% CI, 1.01–1.06; *p* = 0.019). Tumor size-related parameters demonstrated strong associations with prolonged hospitalization, including vertical diameter (OR, 1.07; 95% CI, 1.04–1.11; *p* < 0.001), anteroposterior diameter (OR, 1.13; 95% CI, 1.07–1.18; *p* < 0.001), and transverse diameter (OR, 1.10; 95% CI, 1.05–1.15; *p* < 0.001). In addition, perioperative factors, including anesthesia duration (OR, 1.01; 95% CI, 1.00–1.02; *p* < 0.001) and systolic blood pressure (OR, 1.03; 95% CI, 1.01–1.05; *p* < 0.001), were also independently associated with ELOS. Blood glucose and ACTH8 were not independently associated with ELOS after multivariable adjustment (both *p* > 0.05) and were therefore excluded from subsequent model development.

### 3.3. ROC Curve Analysis and Determination of Optimal Cutoff Values

**Figure 2:**
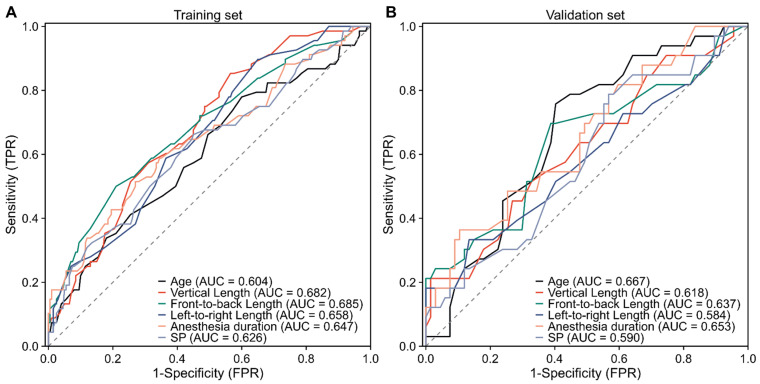
ROC curve analysis for continuous predictors of extended hospital length of stay. (**A**) ROC curves illustrating the predictive performance of variables in the training cohort. (**B**) ROC curves demonstrating the predictive performance of variables in the validation cohort.

ROC curve analysis assessed the discriminative performance of six continuous independent predictors for ELOS and determined optimal cutoffs using the Youden index. As shown in Figure 2, all predictors displayed acceptable discrimination. Anteroposterior tumor diameter yielded the highest AUC (0.685), followed by transverse (AUC = 0.658) and vertical diameters (AUC = 0.682). Age showed modest performance, while anesthesia duration and systolic blood pressure exhibited moderate ability. Table S1 details the metrics: age cutoff >49.5 years (sensitivity 0.78, specificity 0.40, Youden index 0.18); vertical diameter >17.8 mm (sensitivity 0.85, specificity 0.44, Youden index 0.29); anteroposterior diameter >20.5 mm (sensitivity 0.50, specificity 0.80, Youden index 0.30); transverse diameter >17.8 mm (sensitivity 0.90, specificity 0.35, Youden index 0.25); anesthesia duration >193.5 min (sensitivity 0.51, specificity 0.73, Youden index 0.24); and systolic blood pressure >118.5 mmHg (sensitivity 0.66, specificity 0.55, Youden index 0.21). These thresholds dichotomized variables for model integration.

### 3.4. Construction of the Nomogram for Predicting ELOS

**Figure 3:**
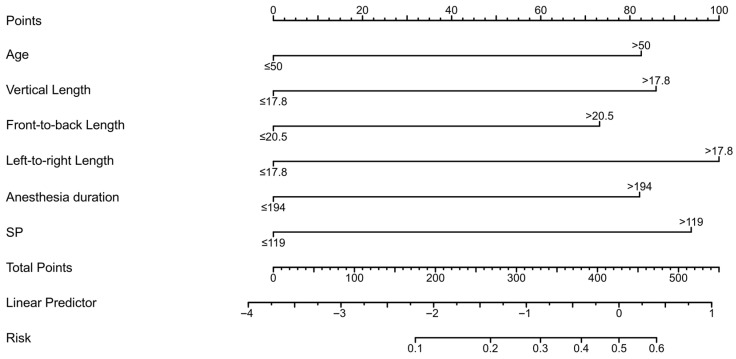
Nomogram for predicting the probability of extended hospital length of stay following endoscopic endonasal transsphenoidal surgery. Abbreviations: SP, systolic blood pressure.

Table S2 lists ORs and 95% CIs for the training cohort (n = 268): age >50 (OR 2.10, 95% CI 1.14–3.85, *p* = 0.017); vertical >17.8 mm (OR 4.47, 95% CI 2.16–9.24, *p* < 0.001); anteroposterior >20.5 mm (OR 3.76, 95% CI 2.10–6.75, *p* < 0.001); transverse >17.8 mm (OR 4.69, 95% CI 2.04–10.81, *p* < 0.001); anesthesia >194 min (OR 2.70, 95% CI 1.53–4.78, *p* < 0.001); systolic >119 mmHg (OR 2.29, 95% CI 1.29–4.04, *p* = 0.004). Similar ORs (1.39–4.63, all *p* < 0.05) were observed in the validation cohort (n = 100). Based on these predictors, the nomogram supports personalized risk assessment for perioperative planning. A nomogram was constructed from multivariable logistic regression-derived predictors to estimate ELOS probability individually. Figure 3 depicts the model incorporating six dichotomized factors: age (>50 years), vertical tumor diameter (>17.8 mm), anteroposterior diameter (>20.5 mm), transverse diameter (>17.8 mm), anesthesia duration (>194 min), and systolic blood pressure (>119 mmHg). Points were assigned based on regression coefficients, with tumor dimensions contributing most to the risk score.

### 3.5. Performance of the Nomogram

**Figure 4:**
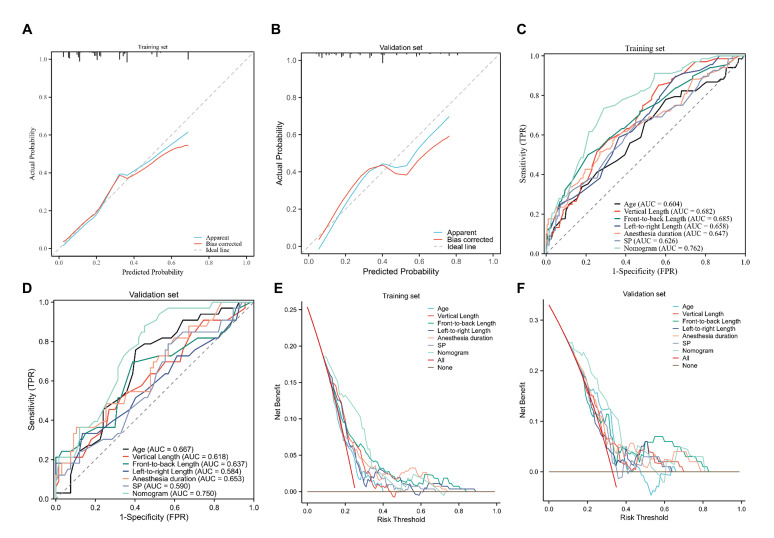
Performance evaluation and external validation of the nomogram for predicting extended hospital length of stay. (**A**) Calibration plot of the nomogram in the training cohort showing agreement between predicted and observed probabilities of ELOS. (**B**) Calibration plot of the nomogram in the external validation cohort demonstrating model reliability. (**C**) Receiver operating characteristic (ROC) curve of the nomogram in the training cohort, demonstrating the discriminative ability of the model. (**D**) ROC curve of the nomogram in the external validation cohort confirming model discrimination. (**E**) Decision curve analysis (DCA) illustrating the clinical net benefit of the nomogram across a range of threshold probabilities in the training cohort. (**F**) Decision curve analysis of the nomogram in the validation cohort showing clinical utility. Abbreviations: ROC, receiver operating characteristic; AUC, area under the curve; DCA, decision curve analysis; ELOS, extended hospital length of stay.

The nomogram demonstrated moderate but reproducible predictive performance. Calibration plots indicated good agreement between predicted and observed probabilities in both cohorts (Figure 4A,B). Calibration was further quantified using the calibration intercept and slope. In the training cohort, the calibration intercept and slope were 0.000 and 1.000, respectively, indicating good apparent calibration. However, the bootstrap bias-corrected calibration curve suggested mild optimism, particularly in the higher predicted-risk range. In the external validation cohort, the corresponding values were 0.149 and 0.720, suggesting acceptable overall calibration with some attenuation of predictions. The nomogram achieves an AUC of 0.762 in the training cohort and 0.750 in the external validation cohort (Figure 4C,D). DeLong’s test in Table S3 confirmed that the nomogram significantly outperformed individual predictors, including age, vertical tumor diameter, and systolic blood pressure, in discriminating ELOS risk (all *p* < 0.05 in the training cohort; *p* < 0.05 for most comparisons in the validation cohort). Furthermore, DCA Figure 4E,F highlighted the model’s potential clinical utility, showing a consistently higher net benefit than “treat-all,” “treat-none,” or single-factor strategies across a broad range of threshold probabilities (approximately 0.10–0.85 in the training cohort and 0.15–0.80 in the validation cohort). These findings support the potential utility of the nomogram as an adjunctive tool for individualized risk stratification rather than as a standalone determinant of clinical decision-making.

### 3.6. Sensitivity Analyses

To assess the robustness of the primary outcome definition, we performed sensitivity analyses using alternative ELOS thresholds based on the 70th and 80th percentiles of LOS in the training cohort. The corresponding thresholds were 14 and 18 days, respectively. Under these alternative definitions, the number of ELOS events in the training cohort was 76 (28.36%) and 60 (22.39%), respectively. Reassuringly, model discrimination changed only minimally, with AUCs of 0.745 and 0.758 in the training cohort and 0.738 and 0.751 in the validation cohort, respectively, supporting the robustness of the primary findings (Table S4).

As an additional sensitivity analysis, we reconstructed the nomogram using the same predictors as continuous variables (Figure S1). The continuous-variable model showed similar discrimination to the primary dichotomized model, with C-indices of 0.778 and 0.771 in the training and validation cohorts, respectively, versus 0.762 and 0.750 for the primary model (Table S5). These findings indicate that the overall predictive performance of the nomogram was not substantially dependent on the dichotomization strategy, although the modestly higher C-indices of the continuous-predictor model suggest some expected information loss after categorization.

## 4. Discussion

In this dual-center retrospective study, we developed and externally validated a nomogram for predicting prolonged hospital length of stay following EETS for NFPAs. By integrating demographic, radiological, and perioperative variables, the model demonstrated moderate discriminative ability, satisfactory calibration, and potential clinical utility in both the training and validation cohorts. These findings suggest that the nomogram may serve as an adjunctive tool for individualized risk stratification and perioperative planning, rather than as a standalone basis for clinical decision-making [25,26].

Age emerged as an independent predictor of extended LOS, consistent with the prior literature [27]. Older patients often present with reduced physiological reserve, a higher burden of comorbidities, and slower postoperative recovery, all of which may contribute to delayed discharge [28]. Additionally, age-related vulnerability to electrolyte disturbances, endocrine fluctuations, and postoperative complications may further prolong hospitalization after pituitary surgery [29]. Tumor size, reflected by vertical, front-to-back, and left-to-right diameters, was also significantly associated with ELOS [30]. Larger tumors are frequently linked to increased surgical complexity, longer operative times, higher risk of cerebrospinal fluid leakage, and greater likelihood of postoperative neurological or endocrinological disturbances [31]. Multidimensional assessment of tumor size, rather than reliance on a single diameter, may therefore provide a more comprehensive representation of surgical burden and recovery risk. Anesthesia duration was identified as another independent risk factor for ELOS. Longer anesthesia time may serve as a surrogate marker for surgical complexity, intraoperative challenges, or technical difficulty [32]. Prolonged anesthesia has also been associated with delayed emergence, increased postoperative nausea and vomiting, and cardiopulmonary stress, which may collectively impede early mobilization and discharge readiness [33,34]. Elevated systolic blood pressure was independently associated with extended LOS [35]. Hypertension may reflect underlying cardiovascular comorbidity and impaired perioperative hemodynamic stability [36,37]. In the context of pituitary surgery, blood pressure fluctuations can complicate intraoperative management and increase the risk of postoperative hemorrhage or electrolyte imbalance, thereby necessitating prolonged monitoring and hospitalization [38].

The nomogram developed in this study offers several potential clinical benefits. First, it enables preoperative and early postoperative identification of patients at high risk for ELOS, facilitating individualized perioperative optimization strategies [39,40]. Second, the model may support more accurate discharge planning and patient counseling, aligning expectations with anticipated recovery trajectories [41]. Third, from a health systems perspective, early risk stratification may improve resource allocation, bed management, and cost containment in high-volume neurosurgical centers [42]. Importantly, the nomogram outperformed individual predictors in terms of discriminative ability, underscoring the value of integrated multivariable risk modeling [43]. The favorable results of decision curve analysis further indicate that the model provides meaningful net clinical benefit across a range of threshold probabilities, supporting its potential utility in real-world practice. Rather than being used as a standalone decision-making tool, it may help identify patients at increased risk of prolonged hospitalization who could benefit from closer postoperative monitoring, earlier discharge planning, more individualized patient counseling, and optimized allocation of beds and perioperative resources. The use of dichotomized predictors in the primary nomogram also warrants consideration. From a statistical perspective, dichotomization may reduce information granularity and predictive efficiency. However, from a clinical perspective, threshold-based predictors are often easier to interpret, communicate, and apply in routine perioperative practice. Because the present nomogram is intended as an adjunctive risk-stratification tool rather than a standalone automated prediction system, clear cutoff values may help clinicians rapidly identify patients who require closer monitoring, earlier discharge planning, or more individualized perioperative management.

Although the absolute duration of hospitalization may vary across institutions and healthcare systems owing to differences in perioperative pathways, discharge criteria, and healthcare delivery models, prolonged LOS remains a clinically meaningful outcome because it generally reflects delayed recovery, increased monitoring needs, greater risk of hospital-associated complications, and higher healthcare resource utilization. Therefore, our intention was not to define a universally fixed LOS threshold applicable to all practice settings, but rather to identify patients at relatively higher risk for extended hospitalization within a given clinical context. In this regard, the percentile-based definition of ELOS was adopted in part to mitigate the influence of inter-institutional variation in absolute LOS. Moreover, the comparable LOS distribution across the two participating centers and the external validation results support the robustness of the model in similar practice environments.

Compared with prior studies on LOS after pituitary surgery, the present work offers several distinctions. First, many previous studies have primarily reported factors associated with prolonged LOS, often in single-center cohorts or heterogeneous pituitary populations, rather than providing a disease-specific and externally validated individualized prediction tool. Second, several prior analyses have relied heavily on postoperative complications, frailty measures, or non-clinical factors, which may be informative for association analysis but are less readily applicable to early individualized perioperative risk stratification. By contrast, our model was specifically developed for patients with non-functioning pituitary adenomas undergoing EETS and integrates routinely available preoperative and perioperative variables into a clinically practical nomogram. Third, the model was developed in a dual-center cohort and externally validated in an independent dataset, which remains relatively uncommon in the current pituitary LOS literature [6]. Therefore, the principal added value of our study lies not in identifying entirely novel predictors, but in translating readily obtainable variables into an individualized risk stratification tool using machine learning that may support earlier perioperative planning in a more homogeneous surgical population [44].

The mean LOS in our cohorts was longer than that reported in some published EETS series, particularly those conducted under enhanced recovery or short-stay discharge protocols. This difference likely reflects local perioperative observation practices, discharge pathways, and healthcare-system factors, in addition to patient- and tumor-related characteristics that may increase postoperative monitoring needs. Therefore, the 14-, 16-, and 18-day thresholds evaluated in this study should not be interpreted as universal benchmarks for prolonged hospitalization after EETS. Rather, these thresholds were used to define relative prolongation of LOS within the present institutional context. Importantly, the mean LOS and ELOS rates were comparable between the two participating centers, suggesting that the observed LOS pattern was relatively consistent within our study setting rather than being driven by a single institutional outlier. Accordingly, the nomogram should be viewed primarily as a risk-stratification tool for comparable practice environments, and further validation is required in institutions with shorter routine LOS, enhanced recovery protocols, or different discharge policies.

Several limitations warrant consideration. First, the retrospective design may introduce selection bias and unmeasured confounding, despite standardized data collection across centers. Second, because hospital length of stay may be influenced not only by patient recovery but also by institutional perioperative pathways and discharge criteria, the absolute LOS values observed in our cohorts may not be directly generalizable to other healthcare systems though the comparable LOS distributions across the two participating centers and the external validation results partially mitigate this concern. Third, several clinically relevant postoperative variables, including complications, extent of resection, and endocrine outcomes, were not included in the present model. This was partly because our aim was to develop an early risk stratification tool based on variables available before or during surgery. Moreover, postoperative cerebrospinal fluid leakage and diabetes insipidus did not occur in either cohort, intracranial infection was rare, and key postoperative variables were not systematically collected across both centers. Although multidirectional tumor dimensions cannot replace formal invasion grading, they may partially reflect tumor burden and operative complexity. Future prospective studies with standardized data collection are warranted to further refine the model [45]. In addition, the comorbidity profile analyzed in the present study was restricted to conditions that were consistently recorded across both centers. Other potentially relevant systemic diseases, including chronic pulmonary, hepatic, or thyroid disorders, were not uniformly available and therefore could not be robustly evaluated. Finally, although the nomogram demonstrated good performance, prospective validation and integration into clinical workflows are required before routine implementation.

## 5. Conclusions

In conclusion, this dual-center study presents a validated nomogram for predicting prolonged hospital length of stay in patients undergoing endoscopic endonasal transsphenoidal surgery for non-functioning pituitary adenomas. By integrating readily available clinical and perioperative factors, the model provides individualized risk estimation and may be useful for perioperative risk stratification and resource planning in similar practice settings. Further prospective multicenter studies are needed to determine its generalizability across different healthcare systems.

## Figures and Tables

**Table 1:** Baseline characteristics of patients with non-functioning pituitary adenomas in the training and validation cohorts.

Variables	Training Cohort (n = 268)	Validation Cohort (n = 100)	Statistic	*p* Value
LOS, mean ± SD	12.50 ± 4.24	12.22 ± 4.16	t = −0.59	0.556
ELOS, n (%)			χ^2^ = 0.03	0.860
No	200 (74.62)	75 (75.00)		
Yes	68 (25.37)	25 (25.00)		
Age, mean ± SD, year	52.30 ± 12.16	50.00 ± 12.34	t = −1.61	0.108
Sex, n (%)			χ^2^ = 0.06	0.799
Female	130 (48.51)	50 (50.00)		
Male	138 (51.49)	50 (50.00)		
Tumor size, mean ± SD, mm				
Vertical	22.36 ± 9.14	22.83 ± 8.42	t = 0.44	0.659
Front-to-back	18.18 ± 6.16	18.44 ± 6.68	t = 0.36	0.720
Left-to-right	21.11 ± 6.60	20.90 ± 6.06	t = −0.28	0.778
LVF, mean ± SD	0.72 ± 0.29	0.75 ± 0.28	t = 0.83	0.409
RVF, mean ± SD	0.74 ± 0.27	0.76 ± 0.27	t = 0.42	0.675
Surgery duration, mean ± SD, min	155.41 ± 50.29	156.77 ± 50.64	t = 0.23	0.818
Anesthesia duration, mean ± SD, min	183.24 ± 52.33	183.78 ± 51.85	t = 0.09	0.930
SP, mean ± SD, mmHg	119.87 ± 15.94	119.09 ± 16.23	t = −0.42	0.678
DP, mean ± SD, mmHg	79.20 ± 10.98	78.38 ± 10.82	t = −0.64	0.522
BMI, mean ± SD, kg/m^2^	25.62 ± 2.01	24.94 ± 3.68	t = −0.40	0.692
Hypertension				
No	230 (85.82)	85 (85.00)	χ^2^ = 0.04	0.842
Yes	38 (14.18)	15 (15.00)		
Diabetes, n (%)			χ^2^ = 0.28	0.595
No	233 (86.94)	89 (89.00)		
Yes	35 (13.06)	11 (11.00)		
Heart disease, n (%)			χ^2^ = 0.77	0.380
No	259 (96.64)	98 (98.00)		
Yes	9 (3.36)	2 (2.00)		
Stroke, n (%)			χ^2^ = 0.00	1.000
No	264 (98.51)	99 (99.00)		
Yes	4 (1.49)	1 (1.00)		
Glucose, mean ± SD, mmol/L	5.34 ± 1.16	5.27 ± 1.01	t = −0.53	0.599
Triglyceride, mean ± SD, mmol/L	2.53 ± 2.38	2.64 ± 2.33	t = 0.41	0.684
Neu, mean ± SD, 109/L	3.49 ± 1.32	3.55 ± 1.63	t = 0.36	0.719
Lym, mean ± SD, 109/L	2.15 ± 0.74	2.20 ± 0.75	t = 0.57	0.570
Mon, mean ± SD, 109/L	0.42 ± 0.18	0.40 ± 0.15	t = −1.04	0.301
Albumin, mean ± SD, g/L	43.83 ± 3.93	44.26 ± 3.97	t = 0.92	0.358
Haemoglobin, mean ± SD, g/L	141.79 ± 16.43	143.74 ± 13.63	t = 1.06	0.292
Platelet, mean ± SD, 109/L	231.88 ± 62.80	244.27 ± 59.08	t = 1.71	0.088
HDL, mean ± SD, mmol/L	1.11 ± 0.34	1.13 ± 0.29	t = 0.59	0.555
LDL, mean ± SD, mmol/L	2.92 ± 1.02	3.08 ± 0.94	t = 1.35	0.178
Cortisol 8, mean ± SD, nmol/L	119.79 ± 67.45	123.16 ± 64.24	t = 0.43	0.666
Cortisol 16, mean ± SD, nmol/L	80.26 ± 65.63	70.91 ± 77.69	t = −1.16	0.249
Cortisol 24, mean ± SD, nmol/L	55.62 ± 68.78	52.39 ± 86.32	t = −0.37	0.710
ACTH 8, mean ± SD, pg/mL	15.99 ± 14.19	18.04 ± 10.64	t = 1.31	0.192
ACTH 16, mean ± SD, pg/mL	7.79 ± 6.63	8.86 ± 14.66	t = 0.96	0.338
ACTH 24, mean ± SD, pg/mL	6.33 ± 5.80	6.06 ± 4.46	t = −0.42	0.674
TSH, mean ± SD, mIU/L	3.00 ± 2.23	3.36 ± 3.67	t = 1.16	0.246
TT4, mean ± SD, nmol/L	68.12 ± 21.33	65.39 ± 24.47	t = −1.05	0.296
TT3, mean ± SD, nmol/L	1.22 ± 0.81	1.26 ± 1.24	t = 0.33	0.742
FT4, mean ± SD, pmol/L	10.75 ± 2.70	10.42 ± 3.07	t = −1.02	0.309
FT3, mean ± SD, pmol/L	2.85 ± 1.14	2.80 ± 0.76	t = −0.44	0.663
GH, mean ± SD, ng/mL	0.14 ± 11.11	0.18 ± 12.44	t = 0.43	0.666
PRL, mean ± SD, ng/mL	21.35 ± 10.05	21.42 ± 11.21	t = 0.39	0.452

This table summarizes demographic, clinical, radiological, and laboratory baseline characteristics of 368 patients, stratified by training (n = 268) and validation (n = 100) cohorts. Continuous variables are expressed as mean ± standard deviation (SD) and compared using Student’s *t*-test (t-statistic). Categorical variables are expressed as counts (percentages) and compared using χ^2^ test or Fisher’s exact test (χ^2^-statistic). *p* values assess differences between cohorts; no significant differences were found (all *p* > 0.05). Abbreviations: LOS, length of stay; ELOS, extended hospital length of stay; LVF, left visual field; RVF, right visual field; SP, systolic blood pressure; DP, diastolic pressure; Neu, neutrophil; Lym, lymphocyte; Mon, monocyte; HDL, high-density lipoprotein; LDL, low-density lipoprotein; ACTH, adrenocorticotropic hormone; TSH, thyroid-stimulating hormone; TT4, total T4; TT3, total T3; FT4, free T4; FT3, free T3; GH, growth hormone; PRL, prolactin.

**Table 2:** Associations between candidate variables and extended hospital length of stay in the training and validation cohorts.

	Training Cohort			Validation Cohort		
Variables	Total (N)	OR (95% CI)	*p* Value	Total (N)	OR (95% CI)	*p* Value
Age	268	1.03 (1.01–1.06)	0.019	100	1.05 (1.01–1.09)	0.015
Vertical tumor size	268	1.07 (1.04–1.11)	<0.001	100	1.06 (1.01–1.12)	0.023
Front-to-back tumor size	268	1.13 (1.07–1.18)	<0.001	100	1.10 (1.03–1.18)	0.005
Left-to-right tumor size	268	1.10 (1.05–1.15)	<0.001	100	1.07 (1.00–1.15)	0.042
Anesthesia duration	268	1.01 (1.00–1.02)	<0.001	100	1.01 (1.00–1.02)	0.005
SP	268	1.03 (1.01–1.05)	<0.001	100	1.03 (1.00–1.05)	0.049
Glucose	268	1.20 (0.96–1.50)	0.118	100	1.13 (0.75–1.70)	0.549
ACTH 8	268	0.98 (0.95–1.01)	0.144	100	1.00 (0.99–1.01)	0.481

This table presents the results of multivariable logistic regression analyses evaluating the associations between selected candidate variables and extended length of stay (ELOS). Data are reported as odds ratios (ORs) with 95% confidence intervals (CIs) and *p* values for each cohort. ORs for continuous variables represent the change per unit increase. Abbreviations: SP, systolic blood pressure; ACTH, adrenocorticotropic hormone.

## Data Availability

Data are available from the corresponding author upon reasonable request.

## Supplementary Materials

The following supporting information can be downloaded at: https://www.mdpi.com/article/10.3390/cancers18101582/s1, Table S1. Optimal cutoff values and diagnostic performance of independent predictors for ELOS in the training cohort; Table S2. Associations between dichotomized independent predictors and extended length of stay in the training and validation cohorts; Table S3. DeLong’s test comparisons of ROC curves between predictors and nomogram in training and validation cohorts; Table S4. Sensitivity analyses of the nomogram using alternative percentile-based definitions of extended hospital length of stay in the training cohort; Table S5. Sensitivity analysis comparing the predictive performance of the primary dichotomized nomogram and the continuous-variable nomogram; Figure S1. Nomogram constructed using continuous predictors for the prediction of extended hospital length of stay. A sensitivity nomogram was reconstructed using the same predictors in their continuous form, including age, vertical tumor diameter, front-to-back tumor diameter, left-to-right tumor diameter, anesthesia duration, and systolic blood pressure.

## Abbreviations

The following abbreviations are used in this manuscript:

EETS	Endoscopic Endonasal Transsphenoidal Surgery
LOS	Length of Stay
ELOS	Extended Length of Stay
NFPA	Non-Functioning Pituitary Adenoma
LASSO	Least Absolute Shrinkage and Selection Operator
ROC	Receiver Operating Characteristic
AUC	Area Under the Curve
C-index	Concordance Index
DCA	Decision Curve Analysis
MRI	Magnetic Resonance Imaging
BMI	Body Mass Index
HDL	High-Density Lipoprotein
LDL	Low-Density Lipoprotein
ACTH	Adrenocorticotropic Hormone
TSH	Thyroid-Stimulating Hormone
T3	Triiodothyronine
T4	Thyroxine
FT3	Free Triiodothyronine
FT4	Free Thyroxine
GH	Growth Hormone
PRL	Prolactin
SP	Systolic Pressure
DP	Diastolic Pressure
LVF	Left Visual Field
RVF	Right Visual Field
Neu	Neutrophil
Lym	Lymphocyte
Mon	Monocyte
