# Supplementary material for: The Prediction of Extended Hospital Length of Stay in Patients After Endoscopic Endonasal Transsphenoidal Surgery for the Resection of Non-Functioning Pituitary Adenomas: A Dual-Center Retrospective Analysis
Source: Cancers (Basel). 2026 May 13;18(10):1582. doi: 10.3390/cancers18101582 (PMC13204605; doi:10.3390/cancers18101582)
Supplement: Supplementary file 1 [file cancers-18-01582-s001.zip › cancers-4255803-supplementary.pdf]

Supplementary materials

Table S1. Optimal cutoff values and diagnostic performance of independent predictors for ELOS in the training cohort.

Variables	Cut-off value	Sensitivity	Specificity	PPV	NPV	Youden index
Age	49.5	0.78	0.40	0.31	0.84	0.18
Vertical tumor size	17.8	0.85	0.44	0.34	0.90	0.29
Front-to-back tumor size	20.5	0.50	0.80	0.45	0.82	0.30
Left-to-right tumor size	17.8	0.90	0.35	0.32	0.91	0.25
Anesthesia duration	193.5	0.51	0.73	0.40	0.82	0.24
SP	118.5	0.66	0.55	0.33	0.83	0.21

Cutoff values were determined using receiver operating characteristic (ROC) curve analysis based on the maximum Youden index. Sensitivity and specificity represent the diagnostic performance of each predictor for identifying prolonged hospital length of stay. Positive predictive value (PPV) and negative predictive value (NPV) were calculated based on the prevalence of prolonged hospitalization in the study cohort. Abbreviations: SP, systolic blood pressure.

Table S2. Associations between dichotomized independent predictors and extended length of stay in the training and validation cohorts.

	Training cohort			Validation cohort		
**Variables**	**Total (N)**	**OR (95% CI)**	***P* value**	**Total (N)**	**OR (95% CI)**	***P* value**
Age	268			100		
≤50	104	Reference		48	Reference	
>50	164	2.10 (1.14 – 3.85)	0.017	52	4.63 (1.82 – 11.78)	0.001
Vertical tumor size	268			100		
≤17.8	97	Reference		26	Reference	
>17.8	171	4.47 (2.16 – 9.24)	< 0.001	74	2.56 (1.07 – 7.55)	0.019
Front-to-back tumor size	268			100		
≤20.5	192	Reference		74	Reference	
>20.5	76	3.76 (2.10 – 6.75)	< 0.001	26	2.16 (1.86 – 5.44)	< 0.001
Left-to-right tumor size	268			100		
≤17.8	77	Reference		32	Reference	
>17.8	191	4.69 (2.04 – 10.81)	< 0.001	68	1.39 (1.06 – 3.49)	0.024
Anesthesia duration	268			100		
≤194	180	Reference		61	Reference	
>194	88	2.70 (1.53 – 4.78)	< 0.001	39	1.80 (1.27 – 4.21)	0.007
SP	268			100		
≤119	135	Reference		48	Reference	
>119	133	2.29 (1.29 – 4.04)	0.004	52	1.40 (1.10 – 3.24)	0.012

This table summarizes the results of multivariable logistic regression analyses examining the associations between dichotomized predictors and extended length of stay (ELOS) in both cohorts. Data are presented as odds ratios (ORs) with 95% confidence intervals (CIs) and P values. Reference categories are indicated for each variable. Abbreviations: SP, systolic blood pressure.

Table s3. Delong's test comparisons of roc curves between predictors and nomogram in training and validation cohorts.

**Cohorts**	**Variable 1**	**Variable 2**	**Statistic**	***P* value**	**Direction**
Training	Age	Nomogram	-3.6815	0.0002	Consistent
	Vertical length	Nomogram	-2.6901	0.0071	Consistent
	Front-to-back length	Nomogram	-2.3838	0.0171	Consistent
	Left-to-right length	Nomogram	-3.1107	0.0019	Consistent
	Anesthesia duration	Nomogram	-2.4231	0.0154	Consistent
	Sp	Nomogram	-3.1583	0.0016	Consistent
Validation	Age	Nomogram	-1.8262	0.0478	Consistent
	Vertical length	Nomogram	-1.8977	0.0477	Consistent
	Front-to-back length	Nomogram	-1.6472	0.0515	Consistent
	Left-to-right length	Nomogram	-2.3736	0.0176	Consistent
	Anesthesia duration	Nomogram	-1.5156	0.0596	Consistent
	Sp	Nomogram	-2.1781	0.0294	Consistent

This table shows delong's test results comparing aucs of individual predictors versus the nomogram for predicting extended length of stay (elos). Negative z-statistics indicate superior nomogram auc. $P < 0.05$ denotes significance. "direction" confirms nomogram outperformance. Abbreviations: sp, systolic blood pressure; roc, receiver operating characteristic; auc, area under the curve.

Table S4. Sensitivity analyses of the nomogram using alternative percentile-based definitions of extended hospital length of stay in the training cohort.

**Outcome**	**Threshold**	**Events in training cohort, n (%)**	**AUC (training)**	**AUC (validation)**
Primary analysis	16 (75th percentile)	68 (25.37)	0.762	0.750
Sensitivity 1	14 (70th percentile)	76 (28.36)	0.745	0.738
Sensitivity 2	18 (80th percentile)	60 (22.39)	0.758	0.751

Extended hospital length of stay (ELOS) was primarily defined as a postoperative length of stay greater than or equal to the 75th percentile of the LOS distribution in the training cohort (⩾16 days). Sensitivity analyses were performed using alternative ELOS thresholds based on the 70th percentile (⩾14 days) and 80th percentile (⩾18 days) of LOS in the training cohort. For each alternative definition, the same predictor set as in the primary model was retained, and model discrimination was re-evaluated in both the training and validation cohorts. Abbreviations: ELOS, extended hospital length of stay; LOS, length of stay; AUC, area under the curve.

Table S5. Sensitivity analysis comparing the predictive performance of the primary dichotomized nomogram and the continuous-variable nomogram.

Model	Predictor format	C-index (training)	C-index (validation)
Primary model	Dichotomized	0.762	0.750
Sensitivity model	Continuous	0.778	0.771

Figure S1. Nomogram constructed using continuous predictors for the prediction of extended hospital length of stay.

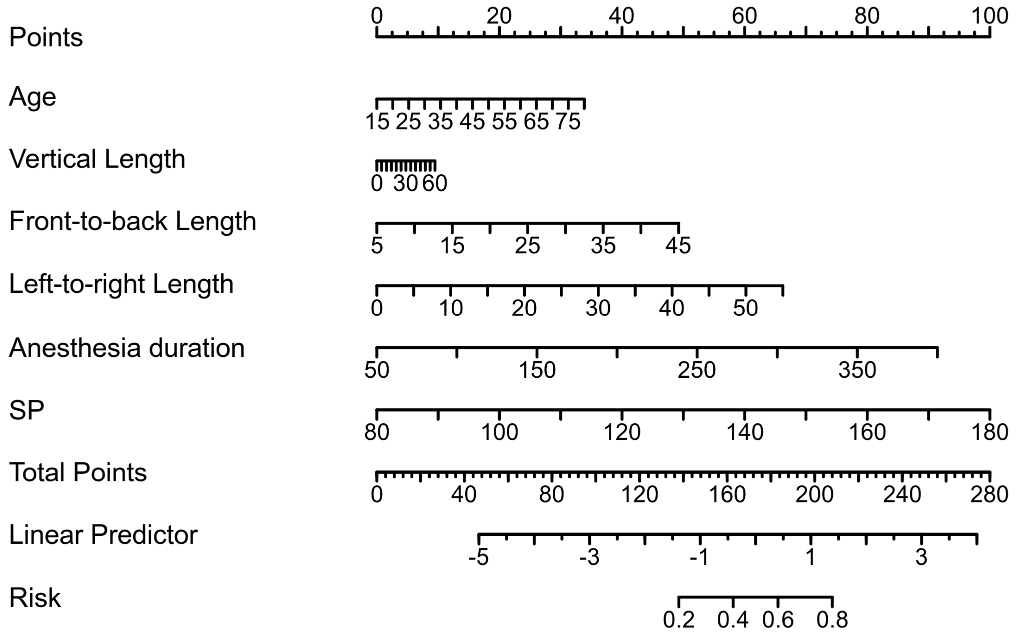

A sensitivity nomogram was reconstructed using the same predictors in their continuous form, including age, vertical tumor diameter, front-to-back tumor diameter, left-to-right tumor diameter, anesthesia duration, and systolic blood pressure.
